# The effects of PPAR-γ agonist pioglitazone on hippocampal cytokines, brain-derived neurotrophic factor, memory impairment, and oxidative stress status in lipopolysaccharide-treated rats

**DOI:** 10.22038/ijbms.2019.36165.8616

**Published:** 2019-08

**Authors:** Farimah Beheshti, Mahmoud Hosseini, Milad Hashemzehi, Mohammad Soukhtanloo, Majid Khazaei, Mohammad Naser Shafei

**Affiliations:** 1 Division of Neurocognitive Sciences, Psychiatry and Behavioral Sciences Research Center, Mashhad University of Medical Sciences, Mashhad, Iran; 2 Department of Basic Science and Neuroscience Research Center, Torbat Heydariyeh University of Medical Sciences, Torbat Heydariyeh, Iran; 3 Iranshahr University of Medical Sciences, Iranshahr, Iran; 4 Department of Biochemistry, Faculty of Medicine, Mashhad University of Medical Sciences, Mashhad, Iran; 5 Neurogenic Inflammation Research Center, Mashhad University of Medical Sciences, Mashhad, Iran; 6 Department of Physiology, Faculty of Medicine, Mashhad University of Medical Sciences, Mashhad, Iran

**Keywords:** Brain-derived neurotrophic- factor, Cytokines learning Lipopolysaccharide, Memory, Pioglitazone

## Abstract

**Objective(s):**

The aim of current study was to evaluate improving effects of pioglitazone as an agonist of peroxisome proliferator-activated receptor gamma (PPARγ), on brain-derived neurotrophic factor (BDNF) and cytokines as well as tissue oxidative damage criteria in the hippocampus in a rat model of lipopolysaccharide (LPS) induced memory impairment.

**Materials and Methods:**

The rats were classified and treated as follows (10 rats per group): (1) vehicle, (2) vehicle before LPS (1 mg/kg, 120 min before memory tests), (3-5) pioglitazone 10, 20 or 30 mg/kg 30 min before LPS. Finally, the hippocampal tissues were collected for biomedical analyses.

**Results:**

In the Morris water maze test, the LPS group, had a longer latency to find the platform while they spent a shorter time in the target quadrant in the probe trial. In the passive avoidance test, the animals of the LPS group had shorter delay times to enter the dark compartment than those of the control group. Treatment with 20 and 30 mg of pioglitazone corrected these parameters. In the hippocampus of LPS group interleukin-6, tumor necrosis factor-α, nitric oxide metabolites, and malondialdehyde were higher while thiol, BDNF, and IL-10 concentrations and the activities of catalase (CAT) and superoxide dismutase (SOD) were lower than the control group. Treatment by both doses of 20 and 30 mg of pioglitazone corrected the biochemical parameters in the hippocampus.

**Conclusion:**

The current findings revealed that pioglitazone protected the rats from learning and memory impairment induced by LPS. The effects were associated with improvement of cytokines, oxidative stress criteria, and BDNF.

## Introduction

Neurofibrillary tangles (NFTs) and senile plaques (SPs) are known as the serious neuropathological signs of Alzheimer’s disease (AD). The creation of SPs and NFTs in different parts of the brain such as the hippocampus, entorhinal cortex, amygdala, and basal forebrain adversely affects learning and memory function (1). In AD patients, a massive loss of synapses, inflammatory processes, and reactive gliosis has been reported to occur in the brain (2). Based on a research conducted on transgenic animals, the neuroinflammatory process has been reported to have a pivotal role in amyloid accumulation in the brain and therefore is considered as a major contributor in AD (3). Additionally, tumor necrosis factor-α (TNF-α) and interleukin (IL)-6 and (IL)-1β cytokines which are produced during inflammation can boost the production of amyloid-beta (Aβ) (4). Interestingly, the beneficial effects of anti-inflammatory drugs on AD have been previously reported (5). It has been thought that inflammatory mediators existing in the brains of AD patients recruit and activate microglial cells and boost some events which leads to an increase in Aβ production (6). Bacterial lipopolysaccharide (LPS) injection, has been well known to be followed by inflammation. After injection, the immune system is activated to promote the production of IL-1, IL-6, and TNF-α cytokines (7). Furthermore, it has been stated that cognitive impairment of mice and rats is induced by intraperitoneal (IP) injection of LPS (6, 8).

Peroxisome proliferator-activated receptor gammas (PPARγ) are a group of ligand-activated transcription factors. They have been well known to control the metabolism of sugars and lipids (9). PPARγ was first proposed as a receptor for the thiazolidinedione drugs (TZDs). These drugs were first known to be a class of insulin-sensitizing medicine and were used to treat type 2 diabetes (10). It has been documented that while TZDs can decrease the generation of superoxide (UO−) and lipid peroxidation, they can increase the activities of SOD and CAT (11-13). Also, it has been reported that PPARγ agonists can inhibit inducible nitric oxide synthase (iNOS) and cyclooxygenase-2 (COX-2) (14, 15). In fact, some examinations have announced the beneficial effects of PPAR γ agonists in neuroinflammatory animal models (15). Thiazolidinedione therapy has been investigated by several studies to treat cognitive deficits concerned with neurodegenerative diseases (16). A great concentration of PPARγ has been identified in the CA1 pyramidal cells of the hippocampus of adult rats and also in the granular and polymorphic layers of the dentate gyrus (17). The fact that TZDs enhance learning and memory functions have been suggested, (18); however, their impacts on learning and memory deficit caused by LPS have not been reported. The current study aimed to evaluate improving effects of pioglitazone as an agonist PPARγ, on the concentration of brain-derived neurotrophic factor (BDNF) and cytokines in the hippocampus as well as hippocampal tissue oxidative damage criteria in a rat model of lipopolysaccharide (LPS) induced memory impairment.

## Materials and Methods

***Drugs and treatments***

Twelve-week old Wistar rats weighing 240–250 g were taken from the Laboratory Animal Center, Faculty of Medicine, Mashhad, Iran. They were kept in a room with a temperature of 21–24 ^°^C and a 12-hr light-dark cycle (lights on at 6 AM). The rats were allowed to use water and food *ad libitum*. Procedures involving the animals and their care were conducted in accordance with the rules set by the Mashhad University of Medical Sciences Ethical Committee (IR.MUMS.fm.REC.1395.186). The rats (n= 50) were classified into the following groups (10 rats per group): (1) Control, (2) LPS, (3) LPS-Pio (pioglitazone) 10 mg, (4) LPS-Pio 20 mg, and (5) LPS-Pio 30 mg. The animals of the LPS, LPS-Pio 10 mg, LPS-Pio 20 mg, and LPS-Pio 30 mg groups were IP injected with LPS (1 mg/kg) 120 min before the memory tests (19, 20). The 3^rd^ to 5^th^ groups were IP injected 10, 20, and 30 mg/kg of the pioglitazone dissolved in saline diluted dimethyl sulfoxide (DMSO), respectively 30 min before the LPS (19). The samples of the control group were injected by 1 ml/kg of vehicle. The powder of LPS had been bought from the Sigma company (Sigma Chemical Co).

***Behavioral evaluation***

*Learning and memory using Morris water maze (MWM)*

The equipment used was a 60 cm-diameter circular pool with a black color. The diameter of the pool was 150 cm and its depth was 60 cm. The pool was filled with water (23–25 ^°^C) to a depth of 30 cm. As explained before (21), the animals were free to find the place of a hidden platform beneath the surface of the water. During the acquisition phase, they underwent four trials per day from different release positions over five days. A rat which had failed to escape onto the platform in 60 sec, was assisted to climb up the platform. A video-tracking system was employed to record the movements of the rats. The latencies and the distances elapsed to reach the platform were obtained from the records. On the 6^th^ day, the platform was removed, and the rats were permitted to look for the maze. Simultaneously, the time spent in the platform area was measured.

*Learning and memory evaluation using passive avoidance test (PAT)*

In this test, a pair of light and dark compartments separated by a removable door forms the training apparatus. A mild electrical shock was applied to the dark part by a stimulator. During two consecutive days and at the beginning of the test, the rats were separately placed in the apparatus (5 min per day) to get accustomed to the environment. On the next day, in order to conduct an acquisition trial, each of the animals was placed inside the light chamber. The opening of the removable door provided the rats with a passage to the dark section. After entering the dark part of the apparatus, a shock (50 Hz and 1.5 mA for 2 sec) was delivered to the animal’s foot. After the shock, the rats were allowed to remain in the dark chamber for an additional 10 sec to keep an association between the place and the shock. Then they were placed in their home cage. Five min later, the rats were again placed in the light chamber to measure short-term memory formation. The training was considered to be completed when the rat remained inside the light chamber for 120 consecutive sec (22, 23). To examine the retention, performances of the rats in PAT were examined at 3, 24, and 48 hr after the acquisition trial. Being free to move around in the two compartments, the rat was placed in the light (safe) section. The delay in entering the dark chamber and the spending time in the dark part were measured for a maximum of 300 sec.

***Biochemical evaluation***

Following the completion of learning and memory tests, we euthanized the rats and removed their brains. Besides, the hippocampus of each rat was collected using an ice-cold plate. The tissues were kept at -80 °C until being utilized. A phosphate buffer (PBS) solution was used to homogenize the tissues. The solutions were centrifuged for 10 min at 1500 rpm. The supernatants were used to measure the malondialdehyde (MDA), total thiol groups, metabolites of nitric oxide (NO), the activity superoxide dismutase (SOD), and catalase (CAT) enzymes. Also, TNF-α, IL-6, and IL-10 cytokines and BDNF were evaluated in the tissues. 

*Evaluation of IL-6, IL-10, TNF-α, and BDNF*

The concentrations of IL-6, IL-10, TNF-α, and BDNF in the hippocampal tissues were determined by ELISA methods and using commercial kits (Ebioscience Co, San Diego, CA, USA), using the instructions provided by the kits. The absorbance of the samples and standards were read using a microplate reader. A standard curve was established using different concentrations of the standard. The absorbance of the samples was compared with the provided standard curve, and the concentrations were obtained. 

*MDA evaluation*

MDA is considered a biomarker of lipid peroxidation. In order to examine the MDA content, the reaction between 2-thiobarbituric acid (TBA) and MDA producing a pink complex with a peak absorbance at 535 nm was measured. Based on the procedure of MDA measurement, which has been previously described (24), 1 ml of the supernatant was combined with a solution containing 2 ml of TBA + trichloroacetic acid (TCA) + hydrochloric acid (HCl). Next, the product was kept in a boiling water bath for 45 min. Eventually, the solution was centrifuged and measured in terms of absorbance at 535 nm.

The following formula was employed to calculate MDA concentration: (C (M) = A /1.65 × 10^5^). 

A: absorbance

Molar absorptivity: 1.65 × 10^5^

*Evaluation of thiol content in the hippocampus*

Based on the Ellman method (25), briefly, 1 ml of tris-ethylenediaminetetraacetic acid (EDTA) buffer was added to 50 µl of each sample solution. The absorbance was measured at 412 nm against tris-EDTA buffer alone (labeled A1). Afterward, 20 μl of DTNB reagent was gathered to A1. The sample absorbance was similarly measured after 15 min (labeled A2). Blank (B) was the absorbance of DTNB. 

Total thiol concentration was achieved as follows: (mM) = (A2-A1-B) × 1.07/0.05 × 13.6

*Measurement of SOD*

The activity of the SOD enzyme was determined considering a method proposed by Madesh and Balasubramanian (26). The method is based on the production of SOD through the auto-oxidation of pyrogallol and dependent inhibition of 3-(4, 5-dimethyl-thiazol-2-yl) 2, 5-diphenyl tetrazolium bromide (MTT) to formazan. The reaction was halted by DMSO. Then each sample’s supernatant was poured into the wells of a 96-well plate. After 5 min, the DMSO was gathered, and the plate was measured with a microplate reader at a wavelength of 570 nm. The amount of protein needed to inhibit 50% reduction of MTT is one unit of SOD.

*Measurement of CAT*

To measure the CAT activity, a blend of 100 µl H2O2 and phosphate buffer at pH 7 was applied for the preparation of the solution used for the measurement (C buffer). Blank was 650 µl of phosphate buffer. The cuvettes for the measurements were filled with the C buffer and sample homogenates. Finally, the decline in the absorption was measured using a spectrophotometer at 240 nm wavelength for 5 min (27).

***Statistical analysis***

All data were reported in the form of mean ± SEM and assessed using the SPSS software (11.5). The 5 day MWA test data and also the data of PAT were analyzed by repeated measures analyses of variance (ANOVA) and Tukey’s *post hoc* test. The MWM test probe day data and biochemical data were compared using one way ANOVA followed by Tukey’s *post hoc* comparisons test. Differences were regarded to be significant in the case of *P*<0.05. 

## Results

***Learning and memory results***

*The MWM results*

**Figure 1:**
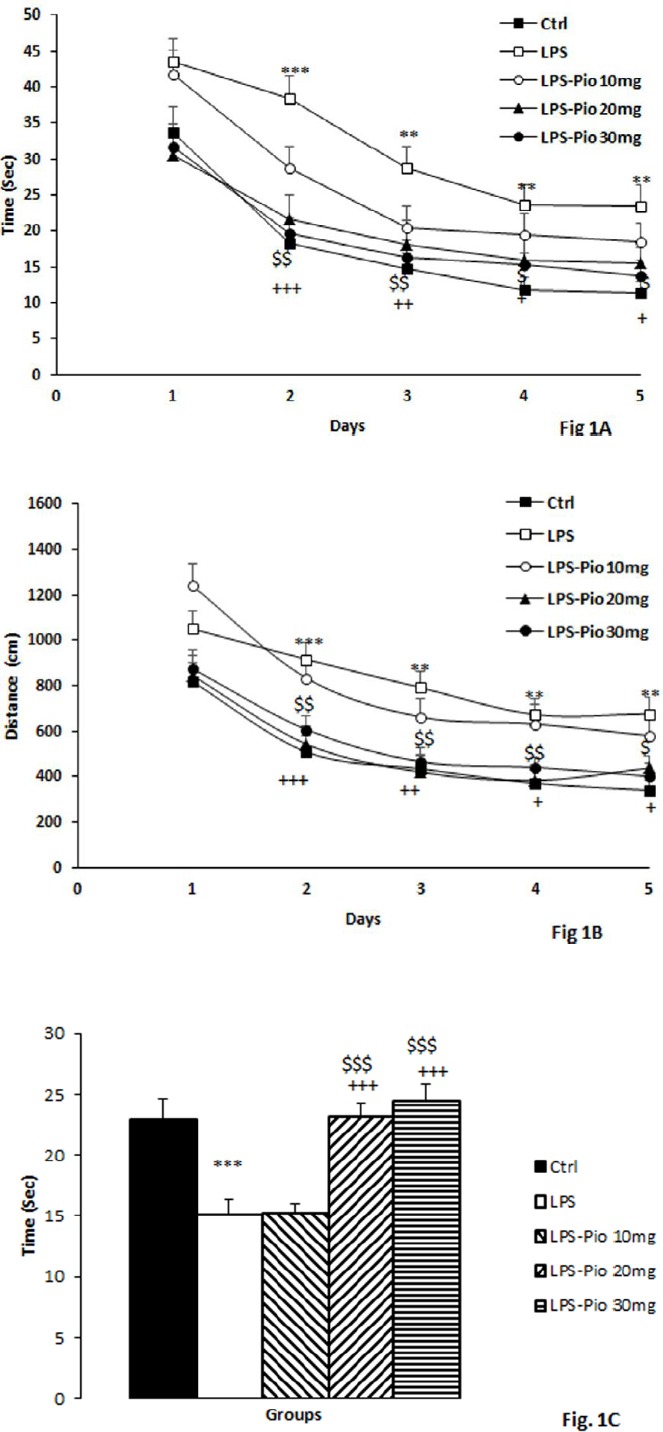
Comparison of time latency (A), path length (B) to reach the platform, and time spent in the target quadrant (C) in MWM test in the experimental groups (Control, LPS, LPS-Pio 10 mg, LPS- Pio 20 mg, and LPS- Pio 30 mg). Data are presented as mean±SEM (n=10 per group). **P<0.01 and ***P<0.001 comparison of LPS with Control group, +*P*<0.05, ++*P*<0.001 and +++*P*<0.001 comparison of LPS- Pio 30 mg with LPS group. $*P*<0.05 and $$*P*<0.01 comparison of LPS- Pio 20 mg with LPS group

The results of repeated measures ANOVA revealed that the day affected the delay time and traveled distance to find the platform (f(4, 975) = 34.094; *P*<0.001 for delay time and f(4, 975) = 37.596; *P*<0.001 for traveled distance). The results also demonstrated a significant effect for the group (f(4, 975) = 19.415, *P*<0.001 for delay time and (f(4, 975) = 20.809, *P*<0.001 for traveled distance). There was no significant interaction between the factors (group×day) f(16, 975) = 0.893, *P*=0.578 for delay time and (f(16, 975) = 0.851, *P*=0.627 for traveled distance) in the 5 days of learning. Furthermore, the *post hoc* test results showed a meaningful difference in the delay time across the 5-day training period between the LPS and the control animals (*P*<0.01-*P*<0.001; Figure 1A). What’s more, traveling distance to find the hidden platform in the animals of the control group was lower than those of the LPS group (*P*<0.01; Figure 1B). Treatment with 20 and 30 mg of pioglitazone reduced delay time and traveled distance within the five days (*P*<0.05-*P*<0.001; Figures 1A and 1B).

Besides, the results of MWM revealed that while being tested in the probe trial, the animals of the LPS group failed to remember the hidden platform location compared to the control animals, the animals of the LPS group spent shorter times in the target quadrant (*P*<0.001; Figure 1C). Treatment with 20 and 30 mg of pioglitazone led to a rise in the time spent in the target quadrant compared with the LPS and LPS-Pio 10 mg groups (*P*<0.001; Figure 1C).

*The results of the PA test*

It was realized from the repeated measures ANOVA that the time after the shock did not affect the delay time (f (2, 135) = 1.536; *P*=0.219). The results also revealed significant effect for group (f (4, 135) = 24.944, *P*<0.001). No significant interaction was observed between the actors (group × the time after the shock) (f (8, 135) = 0.070, *P* = 1.000). Furthermore, the *post hoc* test results showed the subjects of the LPS group entered the dark compartment faster than the ones of the control group at 3, 24, and 48 hr following receiving the shock (*P*<0.001; Figure 1A). Administrating 30 mg/kg Pio before LPS injection caused a greater delay to enter the dark chamber at 3, 24, and 48 hr after the shock relative to that of the LPS group (*P*<0.05; Figure 1A). 

**Figure 2:**
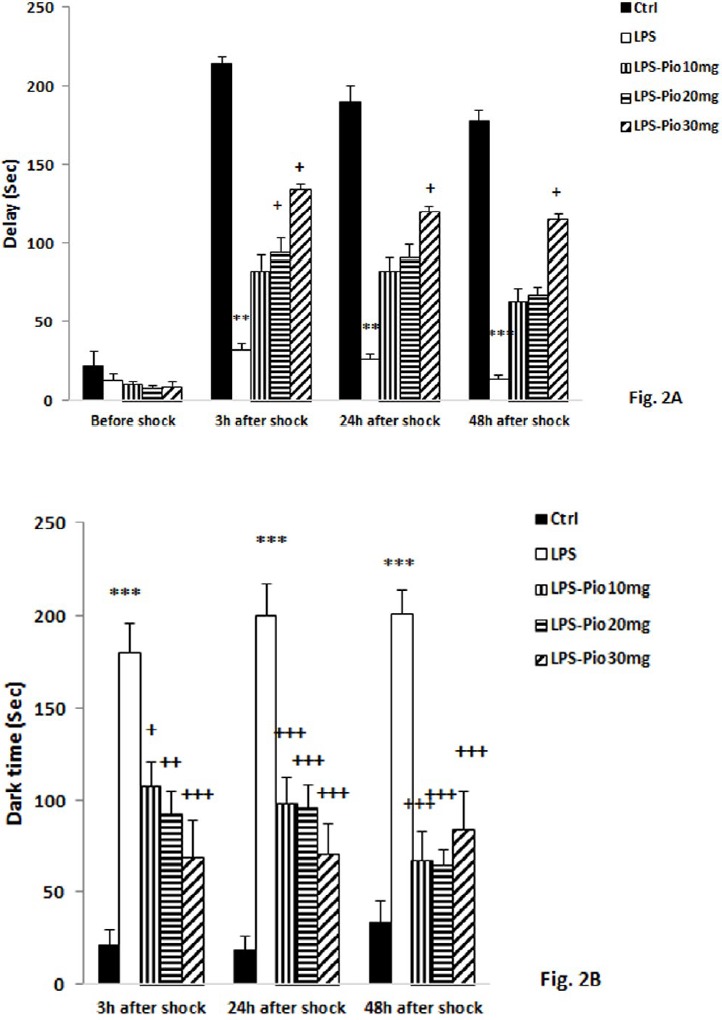
Comparison of the latency for entering the dark compartment (A) and time spent in the dark (B) compartments at 3, 24, and 48 hr after receiving the shock in the experimental groups. Data are presented as mean±SEM (n=10 in each group). **P<0.01 and ***P<0.001 compared to Control group, +*P*<0.05, ++*P*<0.01 and +++*P*<0.001 compared to LPS group

Based on the results of repeated measures ANOVA it was realized that the time after the shock had no impact on the time spent in the dark chamber (f (2, 135) = 0.269; *P*=0.764). Although the results revealed a significant impact for the group (f (4, 135) = 55.047, *P*<0.001), they showed no significant interaction between the parameters (group × the time after the shock) (f(8, 135)= 1.152, *P*=0.333) in the total time spent in the dark chamber. What is more, the *post hoc* test’s results indicated that the time spent in the dark chamber by the rats of the LPS group was higher than that of the control group at 3, 24, and 48 hr after receiving the shock (*P*<0.001; Figure 1B). Treatment by ten, twenty, and thirty mg/ kg Pio before LPS intervention caused a drop in the level of the time spent in the dark compartment at 3, 24, and 48 hr after delivering the shock in comparison with the LPS group (*P*<0.01-*P*<0.001; Figure 2B). 

Pio: pioglitazone; LPS: lipopolysaccharide

Pio: pioglitazone; LPS: lipopolysaccharide; Ctrl: control

**Figure 3:**
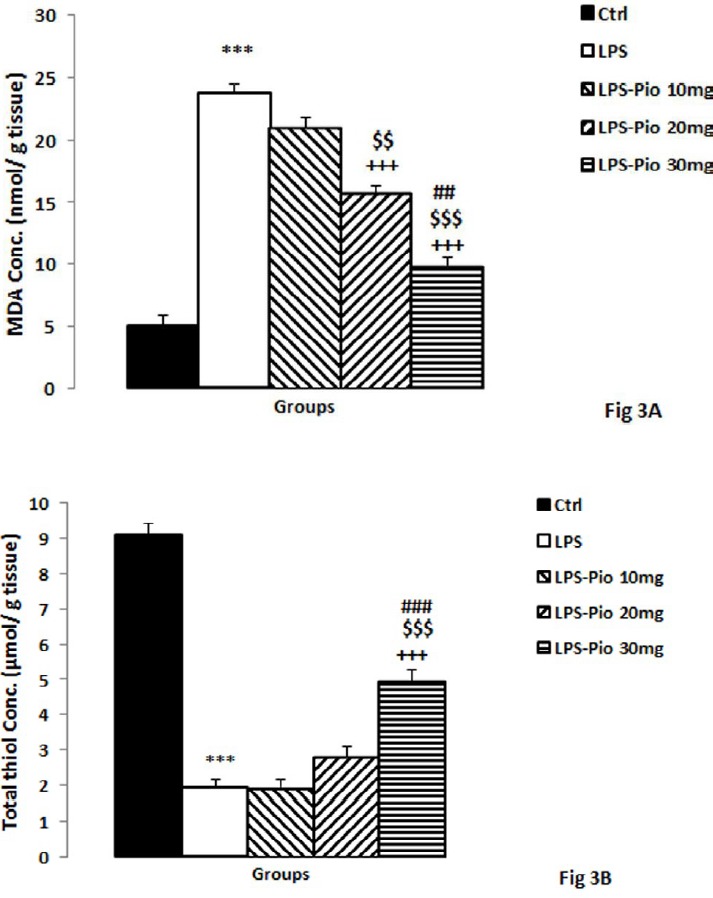
The MDA concentrations (A) and total thiol concentrations (B) in hippocampal tissues among the experimental groups. Data are presented as mean ± SEM (n=10 per group). ***P<0.001 in comparison with Control group, +++P<0.001 in comparison with LPS group. $$$P<0.001 comparison of LPS-Pio 30 mg with LPS-Pio 10 mg groups and ###*P*<0.001 comparison of LPS-Pio 30 mg with LPS-Pio 20 mg groups

Pio: pioglitazone; LPS: lipopolysaccharide; Ctrl: control; MDA: malondialdehyde

**Figure 4:**
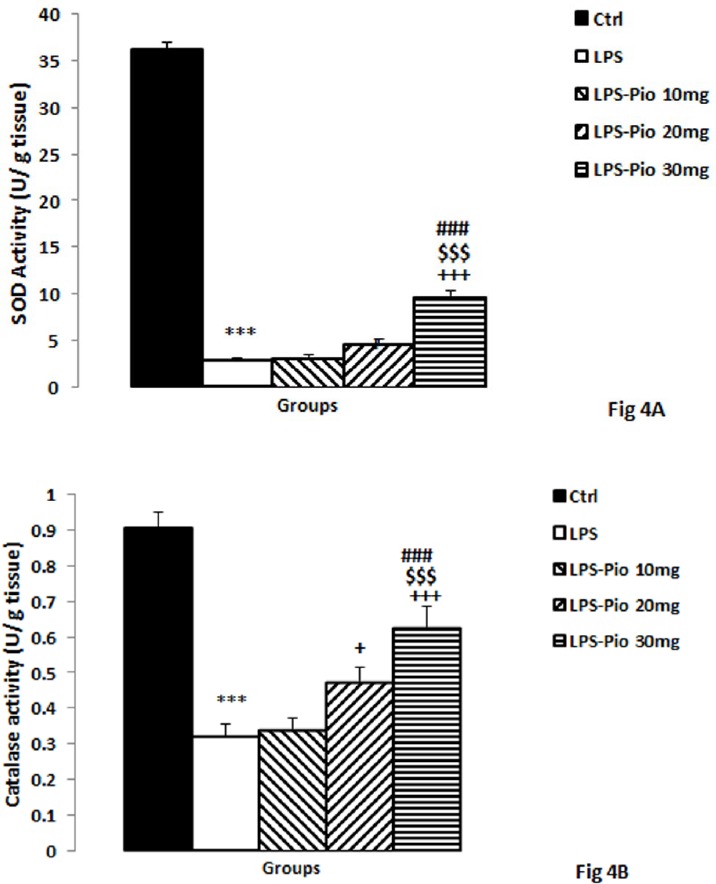
The SOD (A) and CAT (B) activities in hippocampal tissues among the experimental groups. Data are presented as mean±SEM (n=10 per group). ***P<0.001 in comparison with Control group, +*P*<0.05 and +++*P*<0.001 in comparison with LPS group. $$$*P*<0.001 comparison of LPS-Pio 30 mg with LPS-Pio 10 mg group and ###*P*<0.001 comparison of LPS-Pio 30 mg with LPS-Pio 20 mg group

Pio: pioglitazone; LPS: lipopolysaccharide; CAT: catalase; SOD: superoxide dismutase

**Figure 5:**
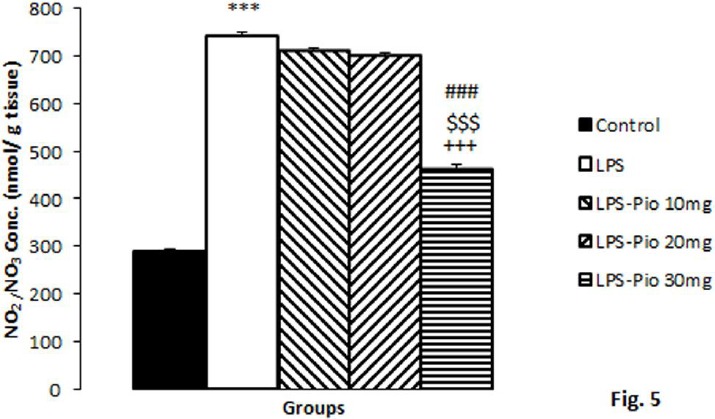
The NO metabolites in hippocampal tissues among the experimental groups. Data are presented as mean±SEM (n=10 per group). ***P<0.001 in comparison with Control group, +++*P*<0.001 in comparison with LPS group. $$$*P*<0.001 comparison of LPS-Pio 30 mg with LPS-Pio 10 mg group and ###*P*<0.001 comparison of LPS-Pio 30 mg with LPS-Pio 20 mg group

Pio: pioglitazone; LPS: lipopolysaccharide; Ctrl: control

**Figure 6:**
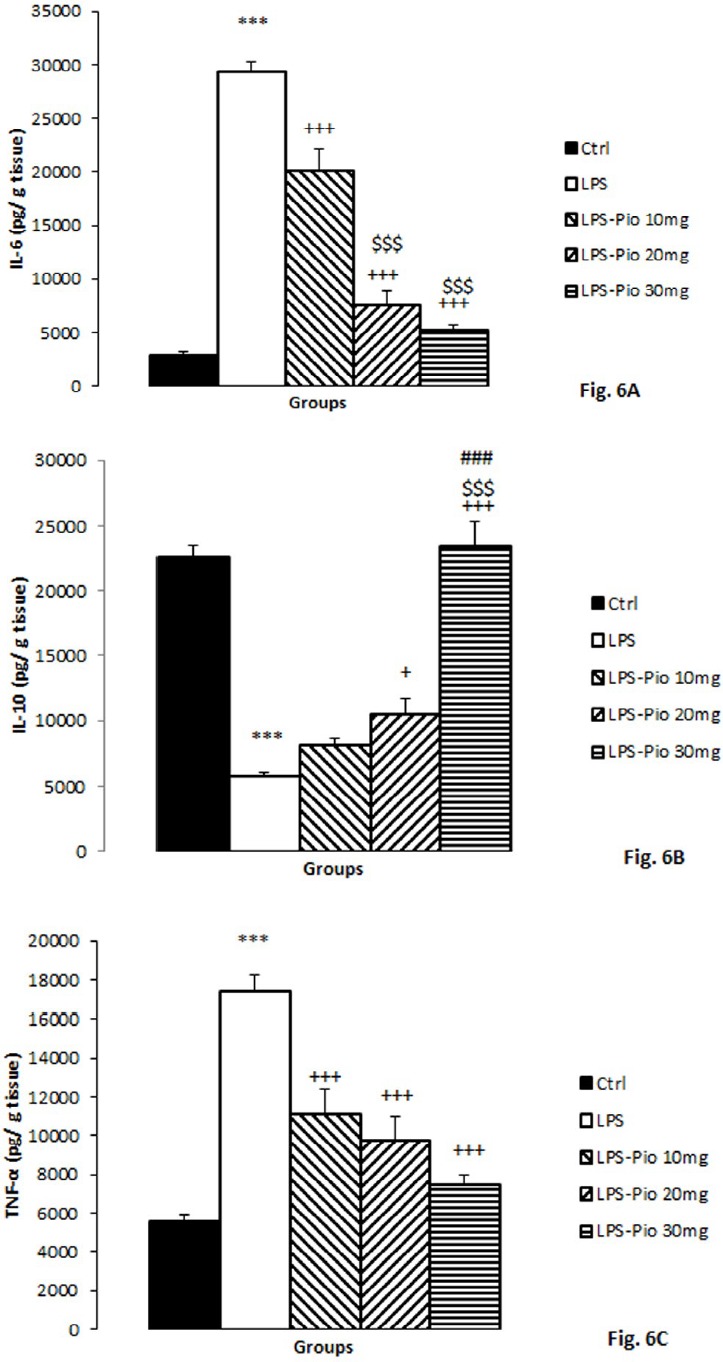
Comparison of IL-6 (A), IL-10 (B), and TNFα (C) among the experimental groups. Data are presented as mean±SEM (n=10 per group). ***<0.001 in comparison with Control group, +*P*<0.05 and +++*P*<0.001 in comparison with LPS group. $$$*P*<0.001 comparison of LPS-Pio 20 mg and LPS-Pio 30 mg with LPS-Pio 10 mg group and ###*P*<0.001 comparison of LPS-Pio 30 mg with LPS-Pio 20 mg group

Pio: pioglitazone; LPS: lipopolysaccharide; Ctrl: control

**Figure 7:**
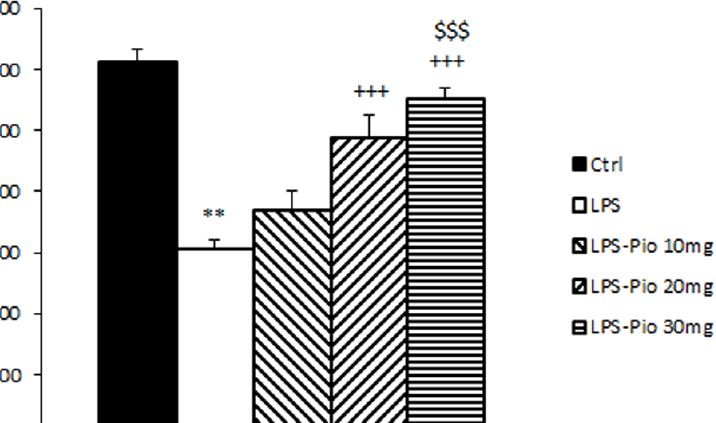
Comparison of BDNF among the experimental groups. Data are presented as mean±SEM (n=10 per group). **P<0.01 in comparison with Control group, +++*P*<0.001 in comparison with LPS group. $$$*P*<0.001 comparison of LPS-Pio 30 mg with LPS-Pio 10 mg group

Pio: pioglitazone; LPS: lipopolysaccharide; Ctrl: control; BDNF: brain-derived neurotrophic factor

***Biochemical results of the hippocampus***

*MDA and thiol concentrations*

Compared to the control animals, the rats of the LPS group had a higher MDA (Figure 3A), but lower thiol concentrations (Figure 3B) (*P*<0.001). A comparison of the MDA results of the groups indicated that administration of 20 and 30 mg/kg Pio reduced hippocampal MDA (*P*<0.001; Figure 3A); conversely, the lowest dose did not have a meaningful impact. Interestingly, in the samples treated with the highest dose of Pio, the MDA level was less than those of the rats treated with 10 and 20 mg of Pio (*P*<0.001; Figure 3A). On top of that, the highest dose of Pio raised the thiol concentration in the hippocampus compared to the LPS group (*P*<0.001; Figure 3B). In addition to that, the highest dose of Pio was shown to be more effective than 10 and 20 mg/kg of Pio in increasing the hippocampal thiol contents (*P*<0.001; Figure 5B).

*Activities of SOD and CAT enzymes*

The results also indicated that the LPS lessened SOD activity in the hippocampus (*P*<0.001). This effect of LPS was prevented by the two higher doses of Pio (*P*<0.05-*P*<0.001). Additionally, SOD of the hippocampus in the animals treated with 30 mg/kg Pio was greater than those of the animals treated with 10 and 20 mg/kg of Pio (*P*<0.001; Figure 4A). 

The results revealed that hippocampal CAT in the LPS-injected animals had a lower activity compared to those of the control ones (*P*<0.001). Although treatment by 10 and 30 mg/kg of Pio boosted the CAT (*P*<0.05-*P*<0.001), 10 mg/kg of Pio did not alter the CAT activity in the hippocampus (Figure 4B). Besides, CAT activity in the hippocampus of the rats treated with 30 mg/kg Pio was higher compared with the ones treated by the lowest and medium doses of Pio (*P*<0.001; Figure 4B).

*Concentration of NO metabolites*

The hippocampal tissue’s NO metabolites in the control group were found to be lower than those of the LPS group (*P*<0.001), though pre-treatment by the highest dose of Pio decreased NO metabolites in the hippocampal tissues (*P*<0.001). Besides, the highest dose of Pio was more effective than the medium (*P*<0.001) and lowest doses (*P*<0.001) (Figure 5). 

*The levels of IL-6, IL-10, and TNF-α*

The IL-6 hippocampal level in the LPS group was higher than that of the control group (*P*<0.001). The results also showed that all doses of Pio (10, 20, and 30 mg/kg) reduced the IL-6 concentration (*P*<0.001; Figure 6A). Additionally, the highest and medium doses of Pio were more implicit than the lowest one (*P*<0.001; Figure 6A). 

The results showed that IL-10 concentration in the hippocampus of the LPS-injected rats was far less than that of the control group (*P*<0.001). Pre-treatment by the two higher doses of Pio (10 and 20 mg/kg) improved IL-10 concentration (*P*<0.05 and *P*<0.001 respectively; Figure 6B). Besides, the highest dose of Pio was the most influential among all doses (*P*<0.001; Figure 6B). 

In the hippocampi of the LPS group, the levels of TNF-α increased compared to the control group (*P*<0.001). Pretreatment by all the doses of Pio (10, 20, and 30) statistically reduced the hippocampal TNF-α by a significant margin. (*P*<0.001; Figure 6C). 

*BDNF content*

The BDNF hippocampal content of the control group was meaningfully higher relative to its value in the LPS group (*P*<0.001). In LPS-Pio 20 and 30 mg-groups BDNF content was higher compared with the LPS group (*P*<0.001; Figure 7). Additionally, it was found that the highest dose of Pio was the most influential one (*P*<0.001; Figure 7).

## Discussion

In the current study, the beneficial properties of Pio on different functions such as learning, memory, oxidative stress criteria, cytokines, and BDNF in the hippocampus of LPS injected rats have been described. LPS is commonly used for induction of animal models of inflammation. This status is usually followed by several actions like mitochondrial malfunction, production of cytokines, and cellular death (28). In the current study, it has been shown that while LPS injection boosted the production of IL-6 and TNF-α, it decreased the level of IL-10. Such alterations are in agreement with the inflammation status in the rats (29). 

In the MWM, the spending time and the traveled distance to find the platform of the LPS group was substantially longer relative to that of the control group. As expected according to the similar findings, it was observed that the time spent in the target quadrant following removal of the platform in the LPS injected rats was lower than the control ones, which indicates that the rats of the LPS group failed to remember the place of the platform. Additionally, in the PAT, a shorter delay was observed to enter the dark section after the shock in the LPS group relative to the control animals. The results of the current study are in agreement with other researchers’ findings: LPS injection causes learning and memory disability in MWM and the Y-maze (30).

According to recent studies, the pivotal role of inflammation in inducing learning and memory impairment is undeniable (31). An increase in the activity of microglia and astrocytes in the brain of LPS-treated animals leads the brain to face injury and the spatial memory to malfunction (32, 33). In this work, LPS has been found to be a contributing factor in learning and memory impairments; a finding associated with an increase in the amount of IL-6 and TNF-α, but a drop in IL-10 of the hippocampus. 

It has been frequently suggested that glial cells activation and the creation of different markers of inflammation, such as IL-1, IL-6, and TNF-α occurs subsequent to the neuro-inflammatory processes. These processes certainly result in neuronal dysfunction in the hippocampus, which is known as inability in learning and memory (33). IL-6 is a mediator with a pro-inflammatory feature. Fundamentally, IL-6 influences leukocytes motivated B-cells to proliferate and secrete antibody and activation of cytotoxic T cells (34). The functions of the macrophage are variously affected by interleukin-10 (IL-10), which is a potent anti-inflammatory cytokine. IL-10 stunningly reduces the TNF-a, IL-8, IL-1, and IL-6 levels. The stimulated macrophages can produce interferon-y (IFN-y) (35). Macrophages can also release reactive oxygen species (ROS), while IL-10 can block this release and prevent the production of NO (36). 

The connection between the brain tissue oxidative damage and deficits of learning and memory has been previously explained (37). In the present study, the results confirmed that LPS –induced neuro-inflammation injection was accompanied with oxidative tissue damage in the hippocampus, which was demonstrated by an increase in the level of NO metabolites and MDA, but a decrease in the level of thiol concentration together with the activities of CAT and SOD enzymes. Similar to our findings, it has been repeatedly shown that LPS-induced neuro-inflammation leads to a rise in the level of MDA while leading to a fall in glutathione, thiols, SOD, and CAT (38-40). Also, LPS reduced the amount of BDNF in the hippocampus. This finding can be another probable mechanism involved in impairing effects of neuroinflammation on learning and memory. Neurotrophins are a class of proteins supporting differentiation and survival of neurons. It is well-understood that the production of these proteins can be affected by the immune system (41,42). In other words, there are some interactions between immune cells and BDNF (4). What is more, it seems that LPS particularly alters the production of BDNF (43). In AD, the reports above seem to be controversial; there are some investigators who have mentioned a modification for BDNF at both mRNA level and protein in both peripheral and the brain tissues (44, 45), suggesting the participation of BDNF in the AD pathophysiology. It has been shown in another study that LPS attenuated the level of BDNF together with other neurotrophins such as NT-3 and nerve growth factor (NGF), which is in agreement with the present results (46). 

Additionally, recent investigations have declared that a huge level of NO production results in a number of biochemistry events that are capable of inducing oxidative stress status, like oxidation of proteins and thiols as well as lipid peroxidation (47). Production of an excessive amount of NO has been regarded as a factor in learning and memory malfunction (48, 49), which has been ascribed to be due to a rise in the activity of iNOS (50). It has been proven that iNOS inhibition results in decreased inflammatory responses, reduced oxidative damage, as well as enhanced learning and memory function (51). Researchers presented that there is a synergistic effect between cytokines such as IL- 6 and TNF-α to release NO from microglial ells (52, 53), while some other researchers have suggested that increased levels of NO are due to activation astrocytes (54, 55).

In the present study, Pio improved performances of rats in both MWM and PAT, which may be considered as its beneficial effects on cognition. In MWM, the rats treated by Pio had a lower delay time to reach the hidden platform and spent a longer time in the target quadrant in comparison to the subjects of the LPS group. Based on our findings in the PA test, delay to enter the dark chamber in the rats treated by Pio was longer compared with the rats of the LPS group. Consistently, it has been previously exhibited that Pio had protective effects against deficits in learning and memory and also against brain tissue oxidative damage (1, 2). According to current research, a high dose of Pio reduced the NO metabolites in the hippocampal region, probably indicating that iNOS has a mediating role in learning and memory improving effects of Pio however, it needs to be challenged in the future. Also, a drop in the hippocampal MDA and a rise in total thiol concentration, CAT, and SOD activities in the Pio-treated subjects revealed that all doses of Pio had protective effects against the hippocampal tissue oxidative damage. 

Also, it has been previously found that injection of Pio attenuated the TNF-α level in the hippocampus (4). With regard to recent studies, it can be implied that in an inflammatory status, glial cells activation leads to the overexpression of iNOS and overproduction of pro-inflammatory cytokines such as TNF-α and IL-1β. The later cytokines are triggering factors for the release of other cytokines, including IL-6. Furthermore, due to increased IL-1β and TNF-α levels, the expression of iNOS in astrocytes and microglia is elevated that is followed by the generation of high levels of NO (56-58). In order to thoroughly address the possible responsible mechanism(s), the effects of all doses including 10, 20, and 30 mg/ kg of Pio were investigated in this experiment followed by measurements of the cytokines levels in the hippocampal region. The results of this study reveal that Pio raises the IL-10 as a well- known anti-inflammatory agent, while it reduces IL-6 and TNF-α as pro-inflammatory markers. Regarding this part of the results, it seems that Pio treatment can prevent the over-activation of glia and release of cytokines in order to enhance the memory function (5). 

It is accepted that BDNF is a beneficial factor in the survival and growth of nerve cells which are vulnerable to AD (59, 60). It has been observed that BDNF improves the viability of neural cells (24) and protects the neurons against neurotoxicity induced by Aβ (61). Regarding these facts, reversion of BDNF to the normal level might be considered as a mechanism for beneficial effects of Pio on learning and memory of the rats, which was seen in the present study. However, it has to be further investigated in future research. Although the exact mechanism(s) of the interaction of Pio with BDNF remains unclear, the association between BDNF and Pio can be suggested as follows: firstly, the hippocampal neurons may be protected by BDNF against neuro-inflammation (62); secondly, BDNF may prevent neurotoxicity mediated by overproduction of NO (63); thirdly, BDNF can regulate the metabolism of free radicals, for example, by an increase in neuronal SOD activity to reduce free radicals (64).

## Conclusion

The results of this paper indicated that Pio had some beneficial effects on learning and memory in an animal model of memory impairment induced by LPS. The effects might be due to a decrease in inflammatory cytokines and protection against hippocampal tissue oxidative damage. Improving anti-inflammatory mediators as well as BDNF may also have a role in the effects of Pio; however, it needs to be challenged more in the future. 
